# Prediction of Axial Capacity of Concrete Filled Steel Tubes Using Gene Expression Programming

**DOI:** 10.3390/ma15196969

**Published:** 2022-10-07

**Authors:** Kaffayatullah Khan, Mudassir Iqbal, Muhammad Raheel, Muhammad Nasir Amin, Anas Abdulalim Alabdullah, Abdullah M. Abu-Arab, Fazal E. Jalal

**Affiliations:** 1 Department of Civil and Environmental Engineering, College of Engineering, King Faisal University, Al-Ahsa 31982, Saudi Arabia; 2 Department of Civil Engineering, University of Engineering and Technology Peshawar, Peshawar 25120, Pakistan; 3 Department of Civil Engineering, University of Engineering and Technology Mardan, Mardan 23200, Pakistan; 4 Department of Civil Engineering, Shanghai Jiao Tong University, Shanghai 200240, China

**Keywords:** concrete filled steel tubes, compression strength, GEP modelling, hyperparameters tuning, parametric and sensitivity analysis

## Abstract

The safety and economy of an infrastructure project depends on the material and design equations used to simulate the performance of a particular member. A variety of materials can be used in conjunction to achieve a composite action, such as a hollow steel section filled with concrete, which can be successfully utilized in the form of an axially loaded member. This study aims to model the ultimate compressive strength (P_u_) of concrete-filled hollow steel sections (CFSS) by formulating a mathematical expression using gene expression programming (GEP). A total of 149 datapoints were obtained from the literature, considering ten input parameters, including the outer diameter of steel tube (D), wall thickness of steel tube, compressive strength of concrete (f_c_’), elastic modulus of concrete (E_c_), yield strength of steel (f_v_), elastic modulus of steel (E_s_), length of the column (L), confinement factor (ζ), ratio of D to thickness of column, and the ratio of length to D of column. The performance of the developed models was assessed using coefficient of regression *R^2^*, root mean squared error RMSE, mean absolute error MAE and comparison of regression slopes. It was found that the optimal GEP Model T3, having number of chromosomes N_c_ = 100, head size H_s_ = 8 and number of genes N_g_ = 3, outperformed all the other models. For this particular model, *R*^2^_overall_ equaled 0.99, RMSE values were 133.4 and 162.2, and MAE = 92.4 and 108.7, for training (TR) and testing (TS) phases, respectively. Similarly, the comparison of regression slopes analysis revealed that the Model T3 exhibited the highest *R^2^* of 0.99 with m = 1, in both the TR and TS stages, respectively. Finally, parametric analysis showed that the P_u_ of composite steel columns increased linearly with the value of D, t and f_y_.

## 1. Introduction

Columns are a desideratum in designing the structural elements of a building. Depending upon the locality, application and resource availability, different materials, for instance, concrete, steel and their combination can be utilized for column construction [1,2,3,4,5]. Reinforced concrete (RC) columns are vastly used around the globe due to a number of reasons, i.e., easy availability of their constituent materials, known behavior under different loading conditions, and development of design codes, such as the ACI 318 [6] design manual and Euro code [7], among others. However, hollow steel sections filled with concrete (HSSFC) represent an improved version in contrast to the other traditional RC columns, both in terms of performance as well as construction costs, alongside possessing the merit of no longitudinal and transverse reinforcement requirements [8]. For example, the hollow steel section (from hereon, referred to as HSS) confines the concrete thus improving its mechanical properties while the buckling resistance of the steel section is enhanced due to the presence of concrete. The composite action of the two materials increases the overall strength, stiffness, ductility, buckling resistance of the element, and provides an improved fire resistance. Since concrete is poured inside the HSS, the need for formwork is eliminated and labor cost is also reduced [9,10,11,12,13].

The cross-sections of the HSS used in concrete-filled hollow steel sections (CFSS) exhibit numerous shapes, i.e., circular, rectangular and square, wherein the most common one is circular because of its high confinement performance [14,15,16]. On a global scale, a number of design codes encompasses the provisions for axial and flexure capacity design of the CFSS [17]. Examples include the Eurocode 4: Design of composite steel and concrete structures (EN1994) [7], AISC-360: Specification for structural steel buildings [18], ACI-318 [6], etc. In addition, the behavior of CFSS has been studied by a number of researchers by considering a number of design parameters such as the slenderness ratio, eccentricity ratio, end-moment ratio, thickness of internal steel tube and compressive strength of concrete, in addition to others [19,20,21], using laboratory experiments and numerical modelling. Although the current codes provide enough guidelines about the axial capacity of CFSS columns, however, uncertainties in the nominal and actual mechanical properties of steel and concrete make the design conservative. In this situation, artificial intelligence (AI) models provide an accurate solution based on mechanical properties of steel and concrete.

Besides the experimental studies for developing knowhow about the influence of various input parameters on the structural performance, soft computing (SC)/machine learning (ML)/(AI)/techniques are gaining popularity nowadays, because of their ability to learn from training data so as to formulate a trained algorithm which can be used for accurate prediction of the output(s) [22,23,24,25]. The accuracy of a typical AI model depends on the number of data points used during the training process and the selection of influential input variables (i.e., high Pearson correlation value). Therefore, several studies have been undertaken recently for evaluating the behavior of CFSS columns under different loading conditions. For example, Albero et al. [26] studied the ultimate resisting load of CFSS under unequal eccentricities at both ends. It was found that the application of unequal load eccentricities enhanced the ultimate resisting load with columns having higher slenderness ratio of 27.78. Similarly, neural networks were successfully used for modelling the strength of columns considering f_v_, f_c_’ and the diameter and wall thickness of the HSS and they concluded that the ANN model successfully predicted the columns’ performance (coefficient of regression *R^2^* > 0.98 in the training, test and validation phases) and recommended it to be a reliable tool for assessing the columns’ performance based on the ANN-based Monte Carlo method [27]. Likewise, the axial load capacity of a CFSS having a circular cross-section was modelled using Artificial Neural Networks (ANN) and Gene Expression Programming (GEP). For example, Naderpour et al. [28] deployed GEP, ANN and group methods of data handling to predict the compressive strength of columns confined with fiber reinforced polymers. It was observed that the ANN model had the highest accuracy among its competitors, with *R^2^* > 0.98. Similarly, the error percentage (± 20% error range) of the forecasted output by ANN, GEP and GMDH was recorded as 94.7, 84.2 and 88.4%, respectively. Similarly, Azim et al. [29] utilized the GEP approach to develop a prediction model for the compressive arch action capacity of RC beam-column substructures. Again, GEP was successfully used for studying the behavior of H-section steel columns (*R^2^* > 0.94) under blast loading by Momeni et al. [30]. In addition, an empirical equation was also generated from the GEP model for relating the damage index to the displacement/rotational index. Wang et al. [31] studied the blast resistance and residual strength of CFSS considering the thickness of steel tube and cross-section geometry. It was observed that the CFSS retained up to 60% of its ultimate axial loading capacity even after close-range blast loading and that their axial load capacity retention was enhanced with the thickness of steel tubes. Similarly, Zhang et al. [32] also concluded that concrete filled steel tubular columns showed excellent performance against flexural loads under both static and dynamic loads.

A literature survey reveals successful application of different AI techniques, such as ANN, GEP and ANFIS alongside the combination of meta-heuristic optimization algorithms and ML algorithms for predicting the mechanical performance of concrete as well as soils [24,33,34,35,36,37,38,39]. However, the ANN and ANFIS provide lesser insight about the models pertaining to their practical implications, e.g., to derive an empirical relation between the input parameters and output(s), which can further be used for predicting the output(s) and performing parametric and sensitivity analysis [40,41]. In addition, both these models are termed as ‘black-box models’ in the literature [40,41], due to the complex interaction of neurons present in different hidden layers. As a result, a useful empirical relation between the input and output parameters is difficult to develop and has reliability issues [42]. In contrast, the white-box models, such as GEP, can provide a simple and easy-to-use mathematical expression to forecast the output for a specific range of input parameters. Using that expression sensitivity and parametric analyses could enable validation of the developed GEP model [43]. Shahmansouri et al. [44] developed an empirical equation for predicting the f_c_’ of geopolymer concrete by considering the specimen’s age, concentration of alkaline activator (i.e., NaOH), natural zeolite, silica fume and blast furnace slag content to be the most influential input parameters. Similarly, Naser et al. [45] developed design equations for the structural response of the CFSS by utilizing the Genetic Algorithm (GA) and GEP technique. They concluded that both GA and GEP models outperformed the current design codes, such as Eurocode 4 [7], AISC 360-16 [18] and New Zealand code (NZS 2327) [46]. Both the models had high predictive capability as a majority of the data points ranged within a 10% bounding error. In an effort to develop an empirical relation between the mix design parameters of lightweight foamed concrete, Sami et al. [47] employed GEP by using 191 data points to develop a model which could simulate the influence of input parameters (i.e., amount of cement, fine aggregate, water to cement ratio and foam volume) on two outputs (i.e., dry density and f_c_’). They revealed that GEP accurately modelled the dry density and f_c_’ of foamed concrete, as evident from their higher values of the coefficient of determination (*R^2^*) (i.e., 0.79 and 0.94, respectively).

In connection to the use of AI models related to predicting the P_u_ of CFSS columns, Sarir et al. [48] used a dataset of 303 points to compare the performance of a GEP model with ANN model optimized by particle swarm optimization (PSO) algorithm, as listed in Table 1. The concrete compressive strength (f_c_’), the column length (L), outer diameter (D), tensile yield stress of the steel column (f_y_), and steel cover thickness (t) were considered as input variables. The best performance was obtained for the GEP model interpreting *R^2^* equaling 0.939. Javed et al. [49] included eccentricities at end supports (e_t_, e_b_) alongside the previously mentioned variables by employing 227 sample specimens. Khan et al. used an extensive database yielding *R^2^* of 0.9812 for test data; however, the value of MAE recorded was comparatively high. Several other researchers (Ngo et al. [50], Jiang et al. [51], Jayalekshmi et al. [52], Ahmadi et al. [53]) also developed Support Vector Regression (SVR) optimized by Grey Wolf Optimization (GWO), GEP, and ANN models, and evaluated the accuracy in terms of *R^2^*. The capabilities of the GEP model were not fully explored by changing its genetic parameters, i.e., number of chromosomes (N_c_), genes (N_g_) and head sizes (h_s_) to obtain the best hyperparameters. Nevertheless, this study utilized 10 variables including the confinement factor (ζ) which was not used in the reported literature.

The ANN and various other such hybrid algorithms (e.g., ANFIS) are unable to provide an empirical equation between the inputs and output(s). As a result, GEP is deployed to robustly simulate the ultimate compressive strength (P_u_) of the CFSS considering different inputs parameters, i.e., (i) outer diameter of steel tube (D), (ii) wall thickness of steel tube (t), (iii) compressive strength of concrete (f_c_’), (iv) elastic modulus of concrete (E_c_), (v) yield strength of steel (f_v_), (vi) elastic modulus of steel (E_s_), (vii) length of the column (L), (viii) confinement factor (ζ), (ix) D/t ratio of column and (x) the L/D ratio of column. The performance of the developed model was assessed using different statistical indices, i.e., *R^2^*, root mean square error (RMSE), mean absolute error (MAE), comparison of regression slopes, predicted to experimental (P/E) ratios. In addition, parametric and sensitivity analyses were also carried out to assess the effect and contribution of input parameters on the P_u_ of CFSS. 

The flow of the paper is that the database is compiled, and the GEP modelling is performed. The formulated GEP model is obtained after undertaking several trials and the effect of genetic parameters on the model performance is studied. After selecting the most optimal trial, the mathematical expression is determined. A variety of performance measures (error indices) were calculated to check the performance of the optimal model. Parametric and sensitivity analyses were also performed for the input parameters considered in the formulated GEP model.

## 2. Methodology

### 2.1. Database Compilation

**Figure 1:**
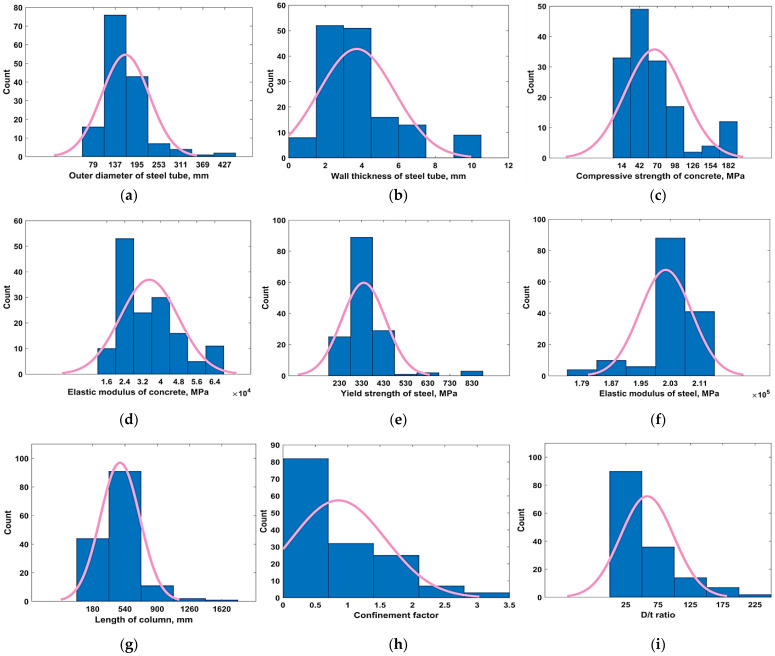

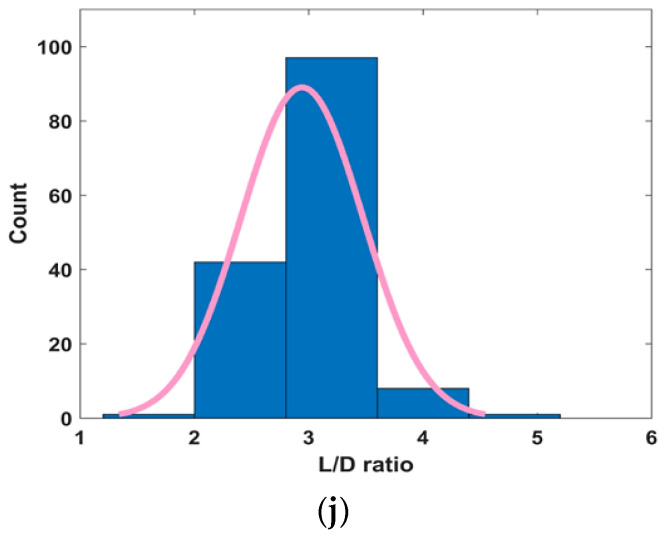
Frequency histograms of input variables. (**a**) Elastic modulus of concrete, (**b**) wall thickness of steel tube, (**c**) compressive strength of concrete, (**d**) Elastic modulus of concrete, (**e**) Yield strength of steel, (**f**) Elastic modulus of steel, (**g**) Length of column, (**h**) Confinement factor, (**i**) D/t, (**j**) L/D.

The database was compiled from the work published by Bardhan et al. [55], which comprises a total of 149 datapoints of which 104 datapoints were used for the training (TR) phase whereas the remaining 49 points were utilized for the testing (TS) phase. Ten input parameters, i.e., D, t, f_c_’, E_c_, f_y_, E_s_, L, ζ, ratio of D and t (i.e., D/t) and the ratio of L and D (i.e., L/D) were considered in order to model their influence on the P_u_ of CFSS using a genetic programming approach. The descriptive statistics of the dataset used for developing the GEP model are listed in Table 2. In order to indicate the frequency distribution of input parameters, the departure of data from the horizontal symmetry (skewness) and the sharpness of the central peak, relative to a standard normal distribution curve [56], the frequency histograms of the input parameters along with their respective normal distribution fit are shown in Figure 1. The values of kurtosis and skewness in Table 2, are in accordance with the aforementioned normal distribution curves. For example, the values of kurtosis and skewness are positive for all the input parameters except for E_c_ and E_s_, respectively. Referring to Figure 1a–c,e–j), the distribution is peaked with a thick tail while Figure 1d shows that the distribution of E_c_ is flatter, which justifies the negative kurtosis value. Similarly, the negative value of skewness for E_s_ can be corroborated from Figure 1a, since the distribution has a fatter tail on its left side.

### 2.2. GEP Modelling

**Figure 2:**
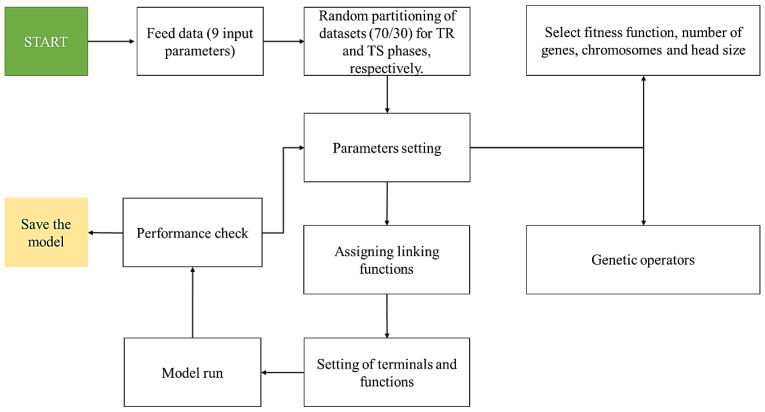
Flowchart of GEP modelling.

GeneXprotools v5.0, developed by Candida Ferreira (Portugal), was employed for developing the desired GEP models. For this purpose, the data was fed into the GEP interface. Afterwards, the dataset was divided into two subsets namely; the TR dataset (70%) and the TS dataset (30%). As a result, 104 datapoints were used for the TR phase whereas the remaining 45 datapoints were used during the TS phase. The option of normalization is readily available in the data tab of this tool; however, the authors did not utilize this option in the current study and the models were trained using the actual values. The authors opine that the statistical models are based upon the descriptive statistics of the given dataset. Similar studies have been widely reported in the past literature [57,58]. In order to achieve the best optimal model, the hyperparameter settings of GEP parameters were adjusted accordingly. For example; the number of chromosomes (N_c_) were varied from 30 to 200, the number of genes (N_g_) from 3 to 5 and, the head size (H_s_) from 8 to 12. Similarly, it was observed that the addition function provides the optimal performance. This was achieved by exploring different linking functions (+, −, ×, /). The details of the genetic parameters settings, i.e., mutation, transposition, and recombination rates are given in Table 3. The details of 11 number of trials employing different values of hyperparameters and the resulting model performance in terms of *R^2^, RMSE* and *MAE* for both the TR and TS phases, respectively, are shown in Table 4. Moreover, Figure 2 shows the flowchart of GEP modelling. The process starts with feeding the input parameters data followed by the random partitioning of datasets. The process is continued by selecting the fitness function, N_c_, H_s_, N_g_ and assigning suitable genetic operators. After assignment of linking functions and terminals setting, the model is run and its performance can be checked using different statistical tools such as *R^2^*, *RMSE* and *MAE*, as shown in Equations (1)–(3), respectively.

(1) $$R^{2}={\left(\frac{\sum_{i=1}^{n}\left(e_{i}-{\bar{e}}_{i})(p_{i}-{\bar{p}}_{i}\right)}{\sum_{i=1}^{n}{\left(e_{i}-{\bar{e}}_{i}\right)}^{2}\sum_{i=1}^{n}{\left(p_{i}-{\bar{p}}_{i}\right)}^{2}}\right)}^{2}$$

(2) $$RRMSE=\frac{1}{\left|\bar{e}\right|}\sqrt{\frac{\sum_{i=1}^{n}{\left(e_{i}-p_{i}\right)}^{2}}{n}}$$

(3) $$MAE=\frac{\sum_{i=1}^{n}\left|e_{i}-p_{i}\right|}{n}$$

where e_i_ and p_i_ are the ith experimental and predicted output values, respectively; ${\bar{e}}_{i}$ and ${\bar{p}}_{i}$ are the average values of the experimental and predicted output values, respectively, and n are the total samples.

A trial and error approach was used for setting the GEP parameters, such that an optimal performing model with the best hyperparameter settings could be obtained. This practice would help avoiding overfitting the data during the TR phase, and, subsequently, improve their performance in the TS phase. In the past, researchers have addressed the problem of overfitting [59]. For example, Gandomi and Roke [60] selected the model with minimum objective function (OF) value which varies from 0 to maximum value, with the model having OF ≈ 0 is considered to have the best performance. Here, statistical evaluation was used to select a non-overfitted model. Table 5 shows the ideal values of the three performance indices, i.e., *R^2^, RMSE* and *MAE*, used to assess the models’ performance.

## 3. Results & Discussion

### 3.1. Variation of Genetic Parameters

Table 4 depicts that a total of 11 trials (Model T1 to T11) were conducted by varying the different parameters of the GEP model (N_c_, H_s_ and N_g_). As discussed earlier, the hyperparameters of GEP models were varied to achieve an optimal GEP model. A total of 11 trials (i.e., Model T1 to T11) were employed with varying values of N_c_, N_g_ and H_s_ (Table 4). Firstly, the values of N_c_ were varied from 30 to 200, while keeping the H_s_ and N_g_ constant (i.e., 8 and 3, respectively). In the second stage, the H_s_ was changed from 8 to 12, while keeping both N_c_ and N_g_ constant. Similar practice was followed for N_g_ as well. The values of N_c_, H_s_ and N_g_ for the best performing model (Model T3) came out to be 100, 8 and 3, respectively. For all the trials, the performance of the models was assessed using *R^2^, RMSE* and *MAE*. It is evident from Table 4 that the model T3 has the highest *R^2^* and, lowest *RMSE* and *MAE*, in both the TR and TS phases, respectively.

**Figure 3:**
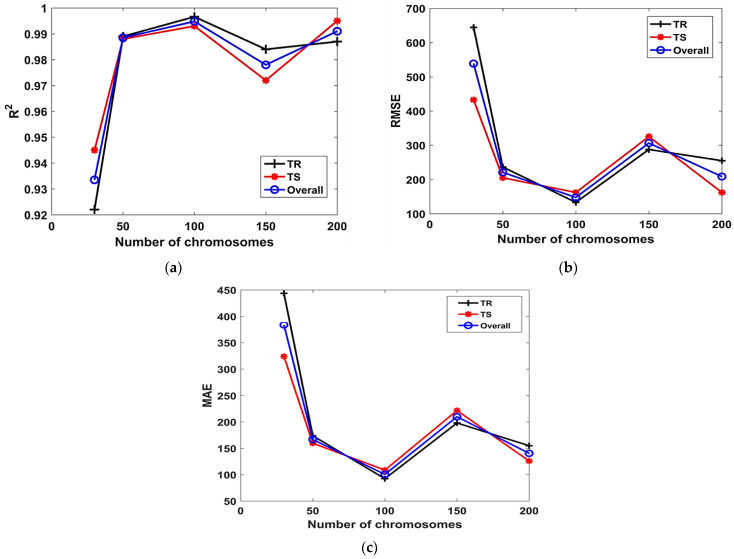
Effect of N_c_ on the performance of models. (**a**) *R^2^*, (**b**) *RMSE*, (**c**) *MAE*.

Figure 3 shows the influence of N_c_ on the *R^2^*, *RMSE* and *MAE* of the models, in both the TR and TS phases, respectively. It can be seen from Figure 3a that, the value of *R^2^* increases with the N_c_ till it drops for N_c_ = 150. A further increase in N_c_ (i.e., from 150 to 200) enhances the *R^2^* again. Similar improvement in the performance of the models with the increase in N_c_ up to 100 can be observed in Figure 3b,c. It is evident from these Figures that the value of *RMSE* and *MAE* plummets with the increase in N_c_; however, the model performs poorly for N_c_ = 150 as evident from its lower *R^2^* = 0.98, 0.97, higher *RMSE* = 287.8, 325.5, and MAE = 198.1, 221.7, in the TR and TS phases, respectively.

**Figure 4:**
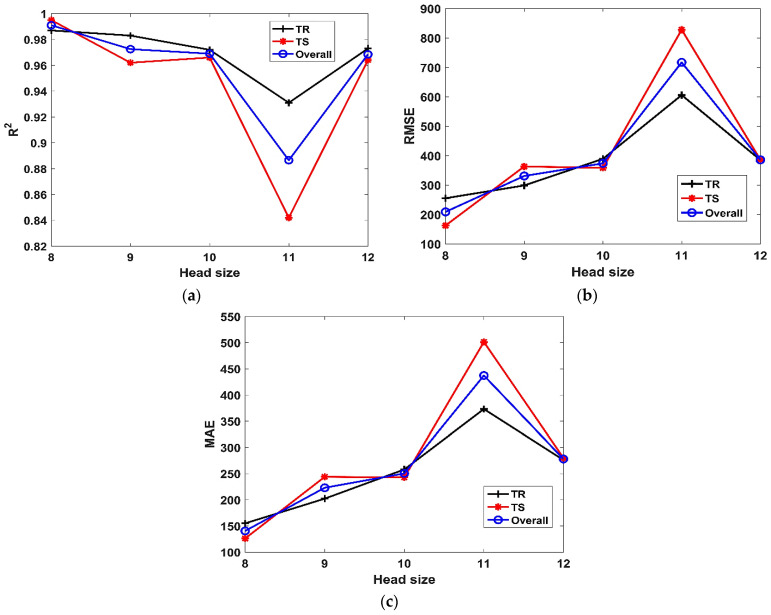
Effect of H_s_ on the performance of models. (**a**) *R^2^*, (**b**) *RMSE*, (**c**) *MAE*.

**Figure 5:**
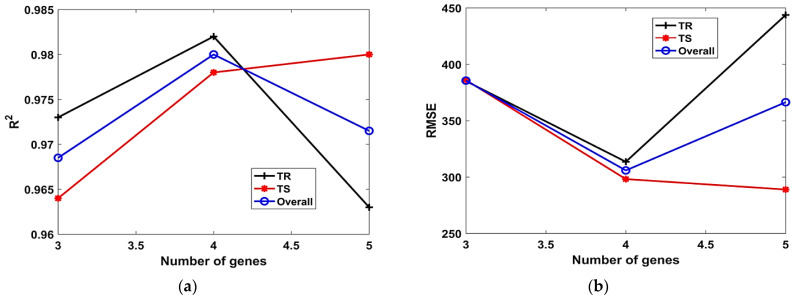

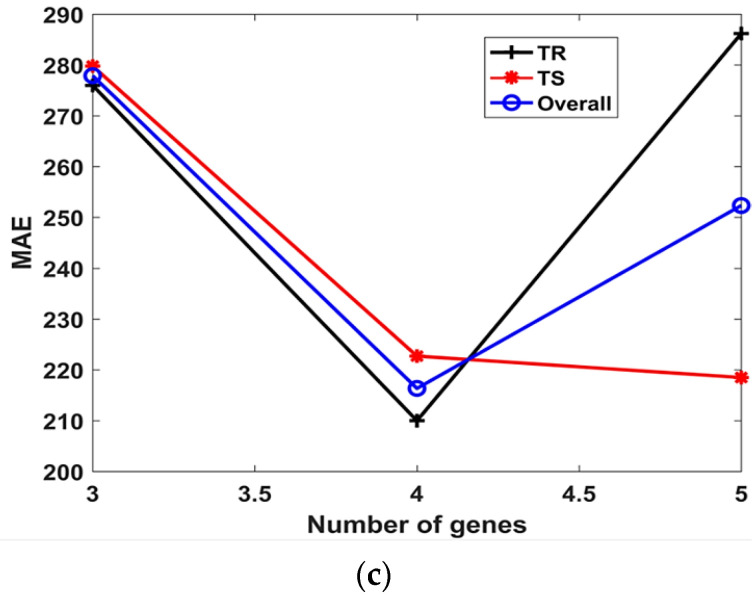
Effect of N_g_ on the performance of models. (**a**) *R^2^*, (**b**) *RMSE*, (**c**) *MAE*.

The influence of H_s_ on the performance of models (both TR and TS phases) in terms of *R^2^, RMSE* and *MAE* can be observed in Figure 4a–c, respectively. It is evident from Figure 4a that, *R^2^* decreases with increasing H_s_. The model depicts immensely poor performance for H_s_ = 11; however, upon further increase in the H_s_ = 12, the value of *R^2^* improves. A similar trend can be observed in the case of Figure 4b,c, wherein the *RMSE* and *MAE* increase with the H_s_. The model performs poorly as is evident from its high *RMSE* and *MAE* values for H_s_ = 11. The influence of N_g_ on the performance of models (TR and TS phases) in terms of *R^2^, RMSE* and *MAE* can be observed in Figure 5a–c, respectively. Considering the TR phase, it is evident from Figure 5a, that the value of *R^2^* increases with the increase in the N_g_ up to 4. Further increase in value of N_g_ (i.e., 4 to 5) lowers the value of *R^2^*. Similar trend can be observed from Figure 5b,c, wherein the value of *RMSE* and MAE decrease with the value of N_g_ (i.e., 3 to 4). When the N_g_ is further increased from 4 to 5, the accuracy of the model decreased as is evident from its high *RMSE* and *MAE* values for N_g_ = 5. However, the performance of the model improves at higher N_g_ in the TS phase, as evident from its higher *R^2^* value = 0.98, and lower *RMSE* and *MAE* values of 288.9 and 218.5, respectively.

### 3.2. Models’ Performance

#### 3.2.1. Statistical Evaluation

It can be seen from Table 4 that the performance of the Model T3 is better (*R^2^* = 0.99 for both the TR and TS phase) followed by Model T5 (*R^2^* = 0.98 in TR phase, and 0.99 for TS phase). The observed values of *R^2^* shows a good agreement between the predicted and experimental values. However, a model cannot be declared as the “best performing model” solely on the basis of *R^2^*. Other statistical error indices must also be considered, such as *RMSE* and *MAE*, in addition to others. In this regard, the values of *RMSE* and MAE were also studied in the current study while assessing the performance of different models. It is evident from Table 4 that, in addition to a higher *R^2^* value, Model T3 exhibited the lowest *RMSE* (133.4 in TR phase, and 162.2 in TS phase) and MAE (92.4 in TR phase and 108.7 in TS phase). Similarly, the Model T5 performed as second-best model having *RMSE* = 255.1 in the TR phase, and, *RMSE* = 478.2, and MAE = 386.6 in the TS phase, respectively. Model T2 also performed better as it possessed the second highest *R^2^* and second lowest value of *RMSE* in the TR phase, respectively. The ranking of the models based on the different statistical indices has also been shown in Table 6.

#### 3.2.2. Comparison of Regression Slopes

Prediction models can be evaluated by plotting a trend line between the experimental and predicted values. This assessment method has also been used in this study and as a result, regression slopes have been plotted for all the 11 models, in both the TR and TS phases, respectively. It is noteworthy to mention that an ideal trend line has a slope “m” value of unity (=1.0) and, its angle of inclination with both the *X*- and *Y*-axis in the cartesian coordinate system equals 45°. The performance of the model is considered to be reliable and accurate provided the plot between the forecasted and experimental values follow the ideal trend line (i.e., inclined at an angle of 45° with the *X*-axis). A regression line whose m value approaches one, and, its correlation value i.e., R ≥0.8 are considered reliable in forecasting new data [61].

**Figure 6:**
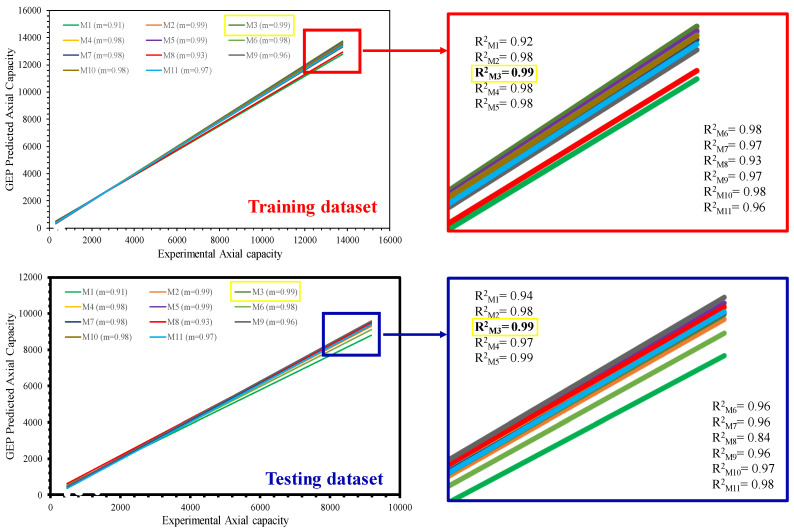
Comparison of regression slopes in the TR phase.

**Figure 7:**
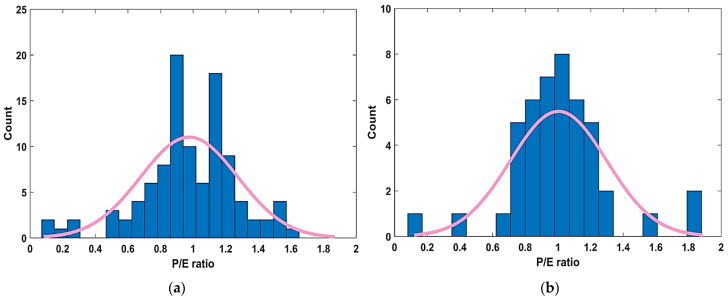
P/E ratio distribution of the best performing model T3, (**a**) TR phase, (**b**) TS phase.

Figure 6 shows the values of *R^2^* and m for both the TR and TS phases, respectively. It is evident from the Figure 6 that *R^2^* exceeds 0.90 for all of the models. In the TR phase, Model T3 has the best fit with an *R^2^* value of 0.99 and m = 1 whereas, Model T1 has a lower value of *R^2^* = 0.92 and m = 0.91. Similarly, the performance of the models improved in the TS phase, as indicated from the higher *R^2^* values and the m values becoming closer to one. All the models have *R^2^* ≥ 0.90. It can also be observed from Figure 6 that, the highest value of m = 1 has been obtained for the optimal model T3 in both the TR and TS phases, respectively. However, Figure 7 shows that in addition to model T3, other models (except T2 and T6) had m values equaling one. It is important to mention here that for m = 1, the slope of the regression line will be exactly 45°. In comparison to other models, the values of ‘m’ and R^2^ observed for the Model T3 are closer to one. Therefore, it can be concluded that the Model T3 is the best performing model, compared to the others.

#### 3.2.3. Model Predicted to Experimental Ratio (P/E)

The performance of the generated models as a result of different trials (by varying N_c_, H_s_ and N_g_) was further studied using the P/E ratio. Figure 7a,b depict the distribution of the P/E ratios for the optimal performing Model T3 in the TR and TS phases, respectively. The bin range has been varied between 0 and 2 using a uniform interval of 0.2. It can be observed from both Figure 7a,b that most of the P/E values (higher frequencies) concentrated in the bin range proximal to one. This serves another statistical check in evaluating the performance of the formulated model and acts as visual justification in case of the optimal performance Model T3.

### 3.3. GEP Formulations

**Figure 8:**
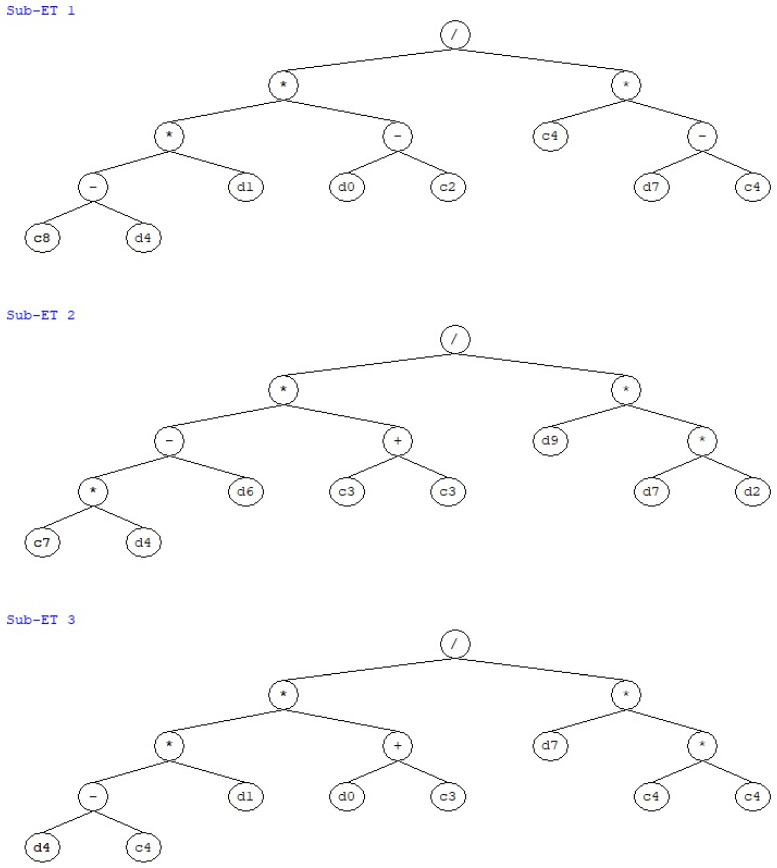
Expression tree for the model T3 depicting the three sub expression trees.

In addition to successful simulation of the P_u_ of CFSS considering a number of input parameters, another novel achievement of this research work is to obtain an empirical equation which can be used for predicting the P_u_ of the CFSS using the different input variables. For this purpose, the optimal performing Model T3 was used to develop the empirical equation. The expression tree for the Model T3 (Figure 8) and the MATLAB model were utilized to obtain the mathematical expression, which can be further used for forecasting the P_u_ of the CFSS, and, sensitivity and parametric analysis can be performed as well. As a result, Equation (4) was obtained, which is able to predict the P_u_ of CFSS using various input variables (i.e., D, t, f_c_’, E_c_, f_v_, E_s_, L, ζ, ratio of D to thickness of column and, the ratio of length to D of column). It is highly recommended to use the prediction equation for input variables whose range and other details have already been discussed in Section 2.1 [62,63].

(4) $$P_{u}=\frac{(-36.9-f_{y})*(D-25.2)*t}{17.2*(\zeta -17.2)}+\frac{((2.3*f_{y})-L)*21.2}{L/D*\zeta *fc’}+\frac{\left((f_{y}-17.2)*t)*(D+2.2\right)}{\zeta *34.4}$$

The values of the constants (c), as present in Figure 8 are as follows:

Sub-ET1: c8 = −36.9; c2 = 25.2; c4 = 17.2; Sub-ET2: c7 = 2.3; c3 = 10.6; Sub-ET3: c4 = 17.2; c3 = 2.2;

Whereas, d0, d1, d2, d3, d4, d5, d6, d7, d8, d9 represents the input parameters such as D, t, f_c_’, E_c,_ f_y_, E_s_, L, ζ, D/t and L/D, respectively.

This Equation (4) is a simple-to-use mathematical expression for designers and practitioners, who can robustly determine the Pu of the CFSS when the easily determinable parameters are available.

### 3.4. Parametric and Sensitivity Analysis

The reliability of different AI models can be verified by conducting parametric analysis of the input features. In this study, the parametric analysis was also carried out for all the input parameters viz., D, t, f_c_’, E_c_, f_v_, E_s_, L, ζ, ratio of D to column, and the ratio of L to D, in order to evaluate their effect on the resulting P_u_ of the CFSS columns. Table 7 shows the possible combination of the study input parameters, considered for the parametric analysis. Hence, a dataset was generated such that one of the input variables (first variable, D) was varied between its extreme values (lower to maximum value in the dataset) in equal increments, while, keeping the remaining input parameters at their average values. In the next step, a second input parameter (i.e., t) was altered in a similar manner, while, keeping the other input features at their mean values. This practice was repeated for all the input parameters. The prediction equation (as given by Equation (1)) was utilized to obtain the corresponding change in the target variable. The range of input variables and their corresponding influence on the P_u_ of CFSS columns was plotted to study their relationship. The net change in the output (P_u_ of CFSS columns) due to changing a particular input feature, was calculated in terms of weighted percentage, in order to find the sensitivity of each input attribute, as well. Note that the sensitivity analysis shows the response of the prediction model by varying the input parameters [64]. The relative contribution of the input parameters can be studied using this analysis as shown in Equation (5) and (6) below.

(5) $$P_{i}=f_{\max}(s_{i})-f_{\min}(s_{i})\times N$$

(6) $$Sensitivity(\%)=\frac{P_{i}}{\sum_{i=1}^{n}P_{j}}\times 100$$

Here, $f_{max}\left(s_{i}\right)$ and $f_{min}\left(s_{i}\right)$ refer to the maximum and minimum GEP estimated values for the i’th input domains, where the remaining input factors = 1. The value of sensitivity analysis is between 0 and 1 which depicts the relative contribution of input parameters among each input attribute as well as the predicted output variable.

**Figure 9:**
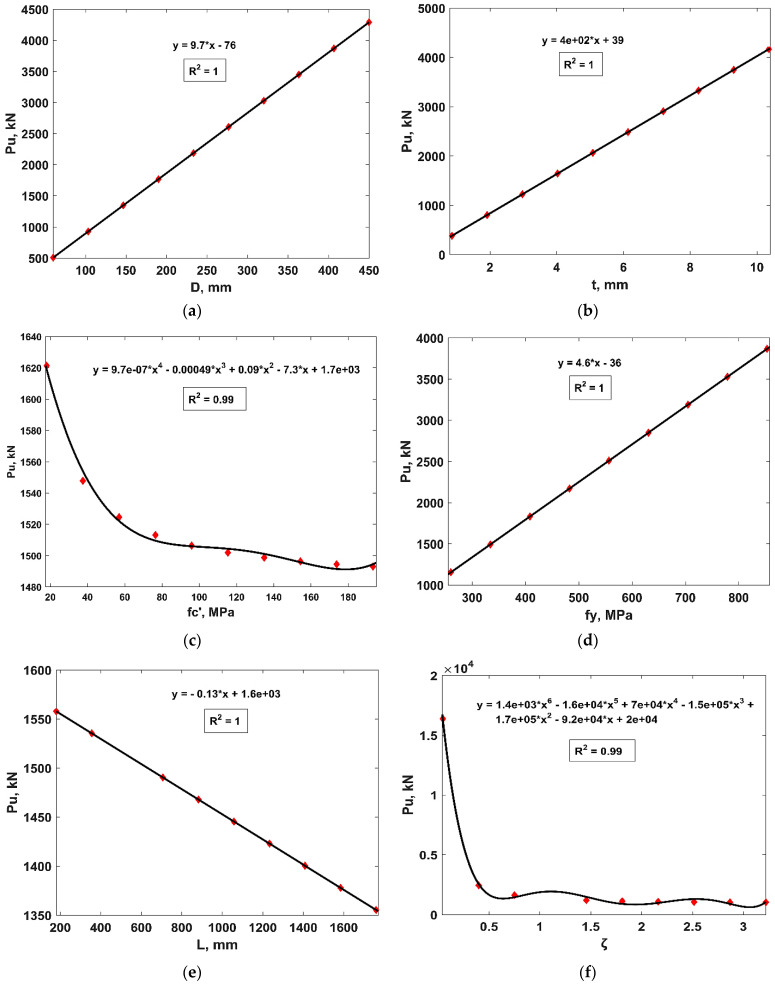

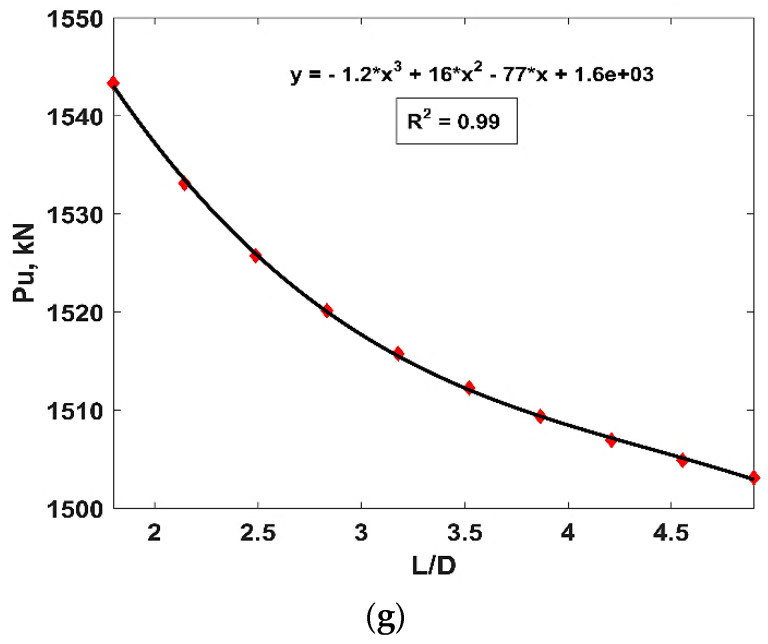
Parametric analysis of input variables. (**a**) Outer diameter of steel tube (D), (**b**) wall thickness of steel tube (t), (**c**) compressive strength of concrete (f_c_’), (**d**) yield strength of steel (f_y_), (**e**) length of column, (L), (**f**) confinement factor (ζ), (**g**) ratio of L/D.

**Figure 10:**
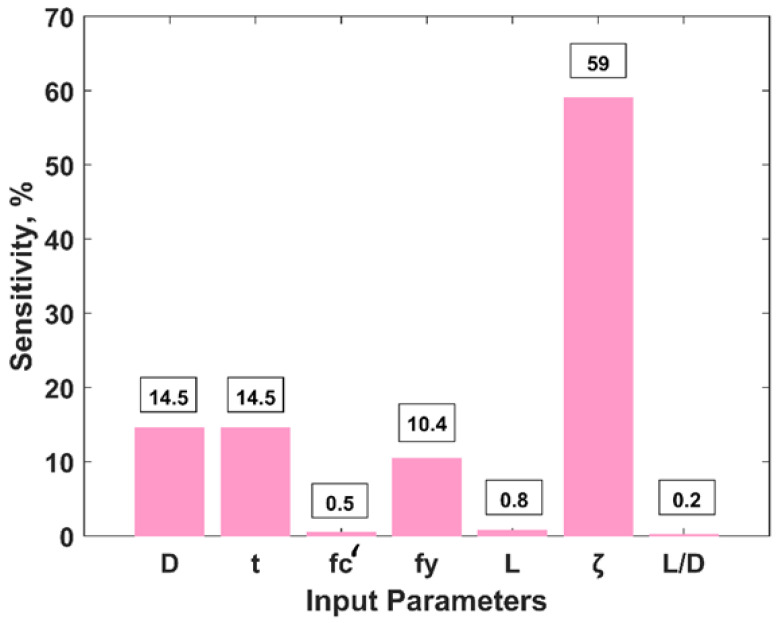
Sensitivity analysis of input variables.

Figure 9 shows the variation of the P_u_ of CFSS columns in response to the change in each input variable, as explained above. It is apparent from Figure 9a,b,d that the P_u_ of CFSS columns increases linearly with the values of D, t and f_y_ while it decreases with the amount of f_c_’, L, ζ and L/D ratio, as is evident from Figure 9c,e–g), respectively. The parametric analysis also revealed that the input parameters, such as E_c_, E_s_ and the ratio of D/t had least influence on the output parameter, i.e., P_u_. Moreover, Figure 9c,f reveal that the value of P_u_ plummets with the increasing values of f_c_’ and ζ. Similarly, different equations such as linear, 3-, 4- and 6-degree polynomial equations were fitted to the resulting parametric analysis, which illustrate good agreement with the datapoints, i.e., all the parameters exhibit *R^2^* ≥ 0.99. The sensitivity analysis of the input variables (as shown in Figure 10) reveals that ζ has the highest value of sensitivity (59%) followed by D and t (14.5%, each). It is also evident that L/D has the least sensitivity value = 0.2. This can be explained from the fact that the confinement of concrete enhances its strength and ductility, which enhances the P_u_. Similarly, increasing value of D and t reduces the slenderness ratio of a column and enables the steel casing to withstand higher tensile stresses imparted by the confined concrete, respectively [21].

## 4. Conclusions

This research study aims to model the ultimate compressive strength (P_u_) of concrete-filled hollow steel sections (CFSS) columns by formulating an empirical relation between the output and input parameters. A total of 149 datapoints were taken from the literature, considering a number of input parameters such as outer diameter of steel tube (D), wall thickness of steel tube, compressive strength of concrete, elastic modulus of concrete (E_c_), yield strength of steel (f_v_), elastic modulus of steel (E_s_), length of the column (L), confinement factor (ζ), ratio of D to thickness of column and the ratio of length to D of column. Different trials were undertaken to develop GEP models using various settings of the hyperparameters.

1. The performance of the developed models was assessed using variety of performance indices, i.e., *R^2^*, *RMSE*, *MAE* and comparison of regression slopes. It was found that Model T3 having N_c_ = 100, H_s_ = 8 and N_g_ = 3 was the optimally performing model among all others. The model exhibits the highest *R^2^* value of 0.99, and the lowest RMSE = 133.4 and 162.2 and MAE = 92.4 and 108.7, in the training and testing phases, respectively.
2. Similarly, the comparison of regression slopes analysis reveals that the Model T3 possess the highest value of *R^2^* equaling 0.99 and m = 1, which represents its high performance and robustness.
3. Finally, the parametric analysis depicts that the P_u_ of CFSS columns increases linearly with the value of D, t and f_y_ while, E_c_, E_s_ and the ratio of D/t had the least influence on the output parameter. The sensitivity analysis of input variables reveals that the ζ is recorded to have the highest value of sensitivity (59%) whereas L/D has the least effect (i.e., 0.2%) in governing the P_u_ of the CFSS.
4. It is highly recommended to use the prediction equation (Equation (4)) for input variables whose range and other details are considered in the descriptive statistics of the current study. The following simple-to-use mathematical expression can be used to predict the P_u_ of the CFSS with higher accuracy.

## Figures and Tables

**Table 1:** Summary of the previous studies in the literature pertaining to prediction of axial capacity of CFSS.

Reference	Model	Number of Data Points	Input Variables	Testing Data Performance	
				*R^2^*	*MAE*
Sarir et al. [48]	GEP	303	f_c_’, L, D, f_y_, t	0.939	_
Javed et al. [49]	GEP	227	f_c_’, L, D, f_y_, t, L/D	0.980	153.9
Khan et al. [54]	GEP	702	f_c_’, L, D, f_y_, t, e_t_, e_b_	0.981	290.36
Ngo et al. [50]	SVR-GWO	802	f_c_’, L, D, f_y_, t, D/t	0.996	_
Jiang et al. [51]	GEP	32	D, L, t, L/D, D/t, f_y_, E_s_, f’c, E_c_, υ	_	_
Jayalekshmi et al. [52]	ANN	633	D, t, f_y_, f_c_’, L	0.962	_
Ahmadi et al. [53]	ANN	272	D, t, f_y_, f_c_’, L	0.801	_
This study	GEP	149	D, t, f_c_’, E_c_, f_y_, E_s_, L, ζ, D/t, L/D	0.99	108.7

**Table 2:** Descriptive statistics of the input variables.

Descriptive Statistics	D (mm)	T (mm)	f_c_’ (MPa)	E_c_ (MPa)	f_y_ (MPa)	E_s_ (MPa)	L (mm)	ζ	D/t (mm/mm)	L/D (mm/mm)
**Average**	164.38	3.71	65.60	3.5 × 10^4^	339.85	201,767	485.07	0.86	58.07	2.94
**Standard Error**	5.17	0.17	3.82	1.1 × 10^3^	8.16	575.81	18.08	0.06	3.38	0.04
**Standard Deviation**	63.09	2.08	46.58	1.3 × 10^4^	99.57	7029	220.73	0.73	41.21	0.53
**Sample Variance**	3980	4.34	2170	1.66 × 10^8^	9914	49,401,978	48,721	1	1698	0
**Kurtosis**	5.18	1.90	0.80	−0.18	11.50	2.21	9.12	0.94	3.03	0.84
**Skewness**	1.79	1.40	1.24	0.83	2.75	−1.01	2.32	1.19	1.78	0.08
**Minimum**	60	0.86	18.03	1.8 × 10^4^	186	177,000	180	0.05	17	1.8
**Maximum**	450	10.36	193.30	6.6 × 10^4^	853	213,000	1760	3.22	221	4.90

**Table 3:** Parameters setting for GEP algorithms.

Parameters	Settings
	Axial Capacity (P_u_) of CFSS
Numerical constants	
Constant per gene	10 Floating number 10 [−10, 10]
Type of data	
Maximum complexity	
Ephemeral random constant	
Genetic operators	
Rate of mutation	0.00138
Inversion rate	0.00546
IS transposition rate	
RIS transposition rate	
1-point recombination rate	0.00277
2-point recombination rate	
Gene recombination rate	
Gene transposition rate	

**Table 4:** Details of the different trials/models conducted to obtain optimal model.

Trial/Model	No. of Variables	No. of Chromosomes	Head Size	No. of Genes	TR Phase			TS Phase		
					*R^2^*	*RMSE*	*MAE*	*R^2^*	*RMSE*	*MAE*
T1	5	30	8	3	0.92	644.4	443.6	0.94	432.9	323.9
T2	7	50	8	3	0.98	236.5	173.8	0.98	205.3	159.6
**T3**	**7**	**100**	**8**	**3**	**0.99**	**133.4**	**92.4**	**0.99**	**162.2**	**108.7**
T4	8	150	8	3	0.98	287.8	198.1	0.97	325.5	221.7
T5	9	200	8	3	0.98	255.1	154.9	0.99	162.3	126.0
T6	7	100	9	3	0.98	298.5	202.1	0.96	363.1	243.8
T7	8	100	10	3	0.97	389.0	257.9	0.96	358.3	242.7
T8	7	100	11	3	0.93	605.8	373.1	0.84	828.0	501.3
T9	8	100	12	3	0.97	385.1	276.0	0.96	386.0	279.8
T10	8	100	8	4	0.98	313.6	209.9	0.97	298.2	222.7
T11	8	100	8	5	0.96	443.8	286.2	0.98	288.9	218.5

**Table 5:** Ideal values of performance indices.

Index	Range/Ideal Value
*R^2^*	(0–1)/1
*RMSE*	(0–∞)/0
*MAE*	(0–∞)/0

**Table 6:** Ranking of models based on *R^2^* and *RMSE*.

Statistic	*R^2^*		*RMSE*		*MAE*	
Rank	1st	2nd	1st	2nd	1st	2nd
TR Phase	T3	T2, T5	T3	T2	T3	T5
TS Phase	T3	T5	T3	T5	T3	T5

**Table 7:** Dataset used for parametric analysis.

Input Variables		Constant Input Parameters	No. of DataPoints
Parameter	Range		
D	60–450	t = 3.71, f_c_’ = 65.60, f_y_ = 339.85, L = 485.07, ζ = 0.86, L/D = 2.94	10
t	0.86–10.36	D = 164.34, f_c_’ = 65.60, f_y_ = 339.85, L = 485.07, ζ = 0.86, L/D = 2.94	
f_c_’	18.03–193.30	D = 164.34, t = 3.71, f_y_ = 339.85, L = 485.07, ζ = 0.86, L/D = 2.94	
f_y_	186–853	D = 164.34, t = 3.71, f_c_’ = 65.60, L = 485.07, ζ = 0.86, L/D = 2.94	
L	180–1760	D = 164.34, t = 3.71, f_c_’ = 65.60, f_y_ = 339.85, ζ = 0.86, L/D = 2.94	
ζ	0.045–3.221	D = 164.34, t = 3.71, f_c_’ = 65.60, f_y_ = 339.85, L = 485.07, L/D = 2.94	
L/D	1.8–4.9	D = 164.34, t = 3.71, f_c_’ = 65.60, f_y_ = 339.85, L = 485.07, ζ = 0.86,	

## Data Availability

The data used in this research have been properly cited and reported in the main text.
